# Supplementary material for: The Prop1-like homeobox gene unc-42 specifies the identity of synaptically connected neurons
Source: eLife. 2021 Jun 24;10:e64903. doi: 10.7554/eLife.64903 (PMC8225392; doi:10.7554/eLife.64903)
Supplement: Supplementary file 1. [file elife-64903-supp1.docx]

**Supplementary File 1: UNC-42(+) neurons and their function**

| UNC-42(+) neuron classes (# of neurons) | UNC-42(+) cells as described by Baran et al., 1999 * | neuron type | Neurotransmitter identity (Reference) | Described function in locomotion  (Reference) | |
| --- | --- | --- | --- | --- | --- |
| ASH (2) | yes | sensory | glutamate (*1*) | sensory inputs induce reversal behavior (*2-10*) | |
| AIB (2) | no ** | interneuron | glutamate (*1*) | backward locomotion (*11*) | |
| AVA (2) | yes | command interneuron | ACh (*12*) | backward locomotion, reversal (*13*) | |
| AVB (2) | no | command interneuron | ACh (*12*) | forward locomotion (*13*) | |
| AVD (2) | yes | command interneuron | ACh (*12*) | backward locomotion (*13*) | |
| AVE (2) | yes | command interneuron | ACh (*12*) | backward locomotion (*13*) | |
| AVH (2) | yes | interneuron | peptidergic |  | |
| AVJ (2) (very dim) | yes |  | ? |  | |
| AVK (2) | yes | interneuron | peptidergic |  |  |
| RIV (2) | yes | interneuron | ACh (*12*) | omega turn (*14*) | |
| RMF (2) | no | ring interneuron | ACh (*12*) |  | |
| SAA (4) | yes | sublateral interneuron | ACh (*12*) |  | |
| RMD (6) | yes | ring motor neuron | ACh (*12*) | long reversal (*14*) | |
| RMH (2) | no | ring motor neuron | ACh (*12*) |  | |
| SMD (4) | no *** | sublateral motor neuron | ACh (*12*) | long reversal, omega turn (*14*) | |
| SIA (4) (very dim) | no | sublateral interneuron | ACh (*12*) |  | |
| SIB (4) | yes | sublateral motor neuron | ACh (*12*) | long reversal (*14*) | |
|  | SMB, AIN, PVT, transient DD, VA11, AS11, VD12, unidentified neurons |  |  |  | |

* We ascribe these differences to misidentification (particularly SMB, AIN) and/or promoter transgene artifacts (PVT) and/or missing regulatory elements in the reporter gene used.

** subsequently found to be expressed in AIB (*11*)

*** subsequently found to be expressed in SMD (*12*)

1. E. Serrano-Saiz *et al.*, Modular Control of Glutamatergic Neuronal Identity in C. elegans by Distinct Homeodomain Proteins. *Cell* **155**, 659-673 (2013).

2. M. A. Hilliard, C. I. Bargmann, P. Bazzicalupo, C. elegans responds to chemical repellents by integrating sensory inputs from the head and the tail. *Curr Biol* **12**, 730-734 (2002).

3. E. R. Troemel, J. H. Chou, N. D. Dwyer, H. A. Colbert, C. I. Bargmann, Divergent seven transmembrane receptors are candidate chemosensory receptors in C. elegans. *Cell* **83**, 207-218 (1995).

4. Y. Sambongi *et al.*, Sensing of cadmium and copper ions by externally exposed ADL, ASE, and ASH neurons elicits avoidance response in Caenorhabditis elegans. *Neuroreport* **10**, 753-757 (1999).

5. A. C. Hart, J. Kass, J. E. Shapiro, J. M. Kaplan, Distinct signaling pathways mediate touch and osmosensory responses in a polymodal sensory neuron. *J Neurosci* **19**, 1952-1958 (1999).

6. D. S. Walker *et al.*, Inositol 1,4,5-trisphosphate signalling regulates the avoidance response to nose touch in Caenorhabditis elegans. *PLoS Genet* **5**, e1000636 (2009).

7. R. Komuniecki, G. Harris, V. Hapiak, R. Wragg, B. Bamber, Monoamines activate neuropeptide signaling cascades to modulate nociception in C. elegans: a useful model for the modulation of chronic pain? *Invert Neurosci* **12**, 53-61 (2012).

8. M. Ezcurra, Y. Tanizawa, P. Swoboda, W. R. Schafer, Food sensitizes C. elegans avoidance behaviours through acute dopamine signalling. *EMBO J* **30**, 1110-1122 (2011).

9. M. Chatzigeorgiou, S. Bang, S. W. Hwang, W. R. Schafer, tmc-1 encodes a sodium-sensitive channel required for salt chemosensation in C. elegans. *Nature* **494**, 95-99 (2013).

10. M. A. Hilliard, C. Bergamasco, S. Arbucci , R. H. A. Plasterk , P. Bazzicalupo, Worms taste bitter: ASH neurons, QUI‐1, GPA‐3 and ODR‐3 mediate quinine avoidance in *Caenorhabditis elegans*. *The EMBO Journal* **23**, 1101-1111 (2004).

11. A. Bhattacharya, U. Aghayeva, E. G. Berghoff, O. Hobert, Plasticity of the Electrical Connectome of C. elegans. *Cell* **176**, 1174-1189 e1116 (2019).

12. L. Pereira *et al.*, A cellular and regulatory map of the cholinergic nervous system of C. elegans. *eLife* **4**, (2015).

13. M. Chalfie *et al.*, The neural circuit for touch sensitivity in Caenorhabditis elegans. *J Neurosci* **5**, 956-964 (1985).

14. J. M. Gray, **J. J. Hill**, C. **I.** Bargmann, A circuit for navigation in Caenorhabditis elegans. *PNAS* **102**, 3184-3191 (2005).
