# Supplementary material for: The Prop1-like homeobox gene unc-42 specifies the identity of synaptically connected neurons
Source: eLife. 2021 Jun 24;10:e64903. doi: 10.7554/eLife.64903 (PMC8225392; doi:10.7554/eLife.64903)
Supplement: Supplementary file 5. [file elife-64903-supp5.docx]

**Supplementary File 5. Strain list.**

| **Strain Name** | **Mutant background** | **Array name** | **DNA on array** | **Reference** | **Footnote** |
| --- | --- | --- | --- | --- | --- |
| OH9382 | *flp-21(ok889)* |  |  | (Consortium 2012) |  |
| LH23 | *lad-2(hd31)* |  |  | (Wang *et al.* 2008) |  |
| VC40759 | *ncam-1(gk798240)* |  |  | (Consortium 2012) |  |
| VC228 | *nlg-1(ok259)* |  |  | (Consortium 2012) |  |
| OH12406 | *ntr-1(tm2765)* |  |  |  |  |
| OH12367 | *ntr-2(tm2243)* |  |  |  |  |
| VC40861 | *rig-1(gk852686)* |  |  | (Consortium 2012) |  |
| RB1364 | *rig-1(ok1540)* |  |  | (Consortium 2012) |  |
| RB1712 | *rig-3(ok2156)* |  |  | (Consortium 2012) |  |
| VH823 | *rig-5(hd48)* |  |  | (Schwarz *et al.* 2009) |  |
| VC884 | *rig-6(gk376)* |  |  | (Consortium 2012) |  |
| VC30212 | *rig-6(gk438569)* |  |  | (Consortium 2012) |  |
| VC40404 | *rig-6(gk621278)* |  |  | (Consortium 2012) |  |
| VC1125 | *rig-6(ok1589)* |  |  | (Consortium 2012) |  |
| CB151 | *unc-3(e151)* |  |  | (Brenner 1974) |  |
| OH13990 | *unc-3(ot839[unc-3::gfp])* |  |  |  |  |
| CB271 | *unc-40(e271)* |  |  | (Brenner 1974) |  |
| CB270 | *unc-42(e270)* |  |  | (Brenner 1974) |  |
| CB419 | *unc-42(e419)* |  |  | (Brenner 1974) |  |
| OH14566 | *unc-42(ot868[unc-42::tagrfp::3Xflag])* |  |  | this work | 9 |
| OH16111 | *unc-42(ot986[unc-42::gfp])* |  |  | this work | 10 |
| NW434 | *unc-6(ev400)* |  |  | (Wadsworth *et al.* 1996) |  |
| CB644 | *unc-62(e644)* |  |  | (Brenner 1974) |  |
| OH13430 | *ceh-20(ok541)* | *muEx261; otIs353* | *muEx261[ceh-20::GFP at C terminus + odr-1::RFP]; otIs353[ric-4fosmid::sl2::nls::yfp::h2b; pha-1(+)]* | (Consortium 2012) |  |
| CB5 | *unc-7(e5)* |  |  | (Brenner 1974) |  |
| CX6161 | *inx-19(ky634)* |  |  | (Chuang *et al.* 2007) |  |
| RB1896 | *inx-18(ok2454)* |  |  | (Voelker *et al.* 2019) |  |
| OH13525 | *inx-6(ot804[inx-6::SL2::NLS::yfp::H2B])* |  |  | (Bhattacharya *et al.* 2019) |  |
| OH16617 |  | *otIs779* | *unc-7fosmid::sl2::nls::yfp::h2b; pha-1(+); myo-2::bfp* | this work |  |
| OH15225 | *pha-1(e2123)* | *otEx7075* | *inx-18afosmid::sl2::nls::yfp::h2b; pha-1(+); myo-2::bfp* | (Bhattacharya *et al.* 2019) |  |
| OH16524 |  | *otIs769* | *inx-19fosmid::sl2::nls::yfp::h2b; pha-1(+); myo-2::bfp* | this work |  |
| CX15758 |  | *kyIs631* | *inx-1p::HisCl1::sl2::gfp* | - (Jin *et al.* 2016) |  |
| NC782 |  | *wdEx290* | *acr-15prom::gfp; unc-119(+)* |  |  |
| OH12318 |  | *otIs491* | *acr-16prom::gfp; rol-6(su1006)* |  |  |
| CZ631 |  | *juIs14* | *acr-2prom::gfp; lin-15(+)* | (Hallam *et al.* 2000) |  |
| OH8908 |  | *otIs251* | *cat-2prom::gfp; rgef-1prom::dsred* |  |  |
| LE311 |  | *lqIs4* | *ceh-10prom::gfp, lin-15(+)* |  |  |
| PD4595 |  | *ccIs4595* | *ceh-24prom::gfp; rol-6(su1006)* |  |  |
| OH16206 | *pha-1(e2123)* | *otEx7442* | *cfi-1prom::gfp; des-2prom::tagrfp +pha-1(+)* | this work | 4 |
| OH16205 | *pha-1(e2123)* | *otEx7441* | *cfi-1prom::gfp; des-2prom::tagrfp; pha-1(+)* | this work | 4 |
| OH16207 | *pha-1(e2123)* | *otEx7443* | *cfi-1prom::gfp; des-2prom::tagrfp; pha-1(+)* | this work | 4 |
| OH12655 |  | *otIs544* | *cho-1fosmid::sl2::nls::mchopti::h2b; pha-1(+)* | (Pereira *et al.* 2015) |  |
| OH10688 |  | *otIs354* | *cho-1fosmid::sl2::nls::yfp::h2b; pha-1(+)* | (Pereira *et al.* 2015) |  |
| OH12496 |  | *otIs518* | *eat-4fosmid ::sl2::nls::mchopti::h2b; pha-1(+)* | (Serrano-Saiz *et al.* 2013) |  |
| OH11124 |  | *otIs388* | *eat-4fosmid::sl2::nls::yfp::h2b; pha-1(+)* | (Serrano-Saiz *et al.* 2013) |  |
| NY2082 |  | *ynIs82* | *flp-12prom::gfp* |  |  |
| OH15621 |  | *otIs704* | *flp-18prom::tagrfp* | this work | 5 |
| OH15622 |  | *otIs705* | *flp-18prom::tagrfp* | this work | 5 |
| NY2054 | *him-5(e1490)* | *ynIs54* | *flp-20prom::gfp* |  |  |
| NY2066 | *him-5(e1490)* | *ynIs66* | *flp-7prom::gfp* | (Kim and Li 2004) |  |
| OH15322 |  | *otIs675* | *flp-7prom::rab-3::gfp::unc-10_3'utr; unc-122prom::gfp* | this work | 2 |
| OH15324 |  | *otIs676* | *flp-7prom::rab-3::gfp::unc-10_3'utr; unc-122prom::gfp* | this work | 2 |
| OH15325 |  | *otIs677* | *flp-7prom::rab-3::gfp::unc-10_3'utr; unc-122prom::gfp* | this work | 2 |
| OH15328 |  | *otIs679* | *flp-7prom::rab-3::gfp::unc-10_3'utr; unc-122prom::gfp* | this work | 2 |
| NC1750 |  | *hdIs32; gvEx173* | *glr-1prom::dsred2; opt-3::gfp + rol-6(su1006)* |  |  |
| OH12052 |  | *otIs476* | *glr-4prom::tagrfp; pha-1(+)* | (Stefanakis *et al.* 2015) |  |
| OH13615 | *pha-1(e2123)* | *otEx6320* | *glr-5fosmid::sl2::nls::yfp::h2b; pha-1(+)* |  |  |
| UL1692 | *unc-119(ed3)* | *leEx1692* | *hlh-34prom::gfp; unc-119(+)* |  | 1 |
| PT2660 |  | *myIs13* | *klp-6prom::gfp* | (Schroeder *et al.* 2013) |  |
| LH247 | *lad-2(tm3056)* | *otEx331* | *lad-2p::GFP + pha-1(+)* | (Wang *et al.* 2008) |  |
| QW84 |  | *zfIs4* | *lgc-55prom::mcherry* | (Pirri *et al.* 2009) |  |
| VH1226 | *pha-1(e2123)* | *hdEx335* | *ncam-1prom::yfp; pha-1(+)* | (Schwarz *et al.* 2009) |  |
| OH15495 |  | *otIs696* | *NeuroPAL* | (Yemini et al., submitted) |  |
| OJ1031 |  | *vjIs47* | *nlg-1prom::gfp* |  |  |
| HA299 | *lin-15B&lin-15A(n765)* | *rtEx222* | *nlp-6prom::gfp; lin-15(+)* |  |  |
| OH15214 |  | *otEx7064* | *nmr-1prom::unc-42::gfp; ttx-3prom::mcherry* | this work | 7 |
| OH15216 |  | *otEx7066* | *nmr-1prom::unc-42::gfp; ttx-3prom::mcherry* | this work | 7 |
| OH15218 |  | *otEx7068* | *nmr-1prom::unc-42::gfp; ttx-3prom::mcherry* | this work | 7 |
| OH15219 |  | *otEx7069* | *nmr-1prom::unc-6::sl2::nls::yfp::h2b; ttx-3prom::mcherry* | this work | 8 |
| OH15220 |  | *otEx7070* | *nmr-1prom::unc-6::sl2::nls::yfp::h2b; ttx-3prom::mcherry* | this work | 8 |
| OH15221 |  | *otEx7071* | *nmr-1prom::unc-6::sl2::nls::yfp::h2b; ttx-3prom::mcherry* | this work | 8 |
| AQ2087 |  |  | *npr-2prom::rfp* |  |  |
| AX1792 |  | *dbEx721* | *npr-4prom::mcherry; unc-122prom::gfp* | (Cohen *et al.* 2009) |  |
| OH15369 |  | *otIs680* | *npr-9prom::rab-3::gfp::unc-10_3'utr; unc-122prom::gfp* | this work | 2 |
| OH15370 |  | *otIs681* | *npr-9prom::rab-3::gfp::unc-10_3'utr; unc-122prom::gfp* | this work | 2 |
| OH12467 |  | *otEx5646* | *ntc-1fosmid::sl2::nls::mchopti::h2b; rol-6(su1006)* |  |  |
| OH12750 |  | *otIs547* | *ntr-1prom::gfp; elt-2prom::mcherry* |  |  |
| OH12486 |  | *otEx5659* | *ntr-2fosmid::sl2::nls::yfp::h2b; rol-6(su1006)* |  |  |
| LX990 | *lin-15B&lin-15A(n765)* | *vsEx494* | *ocr-2prom::gfp::ocr-2_3'utr; lin-15(+)* |  |  |
| CX3260 |  | *kyIs37* | *odr-10prom::gfp, lin-15(+)* | (Sengupta *et al.* 1996) |  |
| OH11809 |  | *otIs450* | *oig-1fosmid::sl2::gfp; rol-6(su1006)* | (Howell *et al.* 2015) |  |
| HA0003 |  | *nuIs11* | *osm-10prom::gfp; lin-15(+)* | (Hart *et al.* 1999) |  |
| PY6100 |  | *oyIs59* | *osm-6prom::osm-6::gfp* |  |  |
| OH14456 | *ot845[bnc-1::mneongreen::3xflag::aid])* | *otEx6773* | *pdf-1prom::nls::mchopti::h2b; myo-2prom::mcherry* |  |  |
| LSC87 |  | *lstEx6* | *pdf-2prom::gfp; myo-3prom::mcherry* | (Janssen *et al.* 2009) |  |
| PT2351 | *him-5(e1490)* | *myEx741* | *pdfr-1prom::nls::rfp; unc-122prom::gfp* | (Barrios *et al.* 2012) |  |
| GD387 |  | *ivIs26* | *phat-1prom::wcherry; glr-2prom::gfp; rol-6(su1006)* |  |  |
| OH10689 |  | *otIs355* | *rab-3prom::nls::tagrfp* | (Stefanakis *et al.* 2015) |  |
| VH1432 |  |  | *rig-1prom::yfp* | (Schwarz *et al.* 2009) |  |
| OH4326 | *pha-1(e2123)* | *otEx239* | *rig-3prom::GFP; pha-1(+)* | (Aurelio *et al.* 2002) |  |
| OH12831 | *pha-1(e2123)* | *otEx5883* | *rig-5prom::rig-5::sl2::gfp; pha-1(+)* | (Vidal *et al.* 2015) |  |
| VH1223 | *pha-1(e2123)* | *hdEx332* | *rig-5prom::yfp; pha-1(+)* | (Schwarz *et al.* 2009) |  |
| VH1224 | *pha-1(e2123)* | *hdEx333* | *rig-6prom::yfp; pha-1(+)* | (Schwarz *et al.* 2009) |  |
| OH1422 |  | *otIs138* | *ser-2prom::gfp; rol-6(su1006)* |  |  |
| OH15625 |  | *otIs708* | *sra-11prom::wrmscarlet; inx-6prom::tagrfp* | this work | 6 |
| FG802 |  |  | *srd-10prom::gfp* |  |  |
| OH15173 |  | *otIs663* | *srd-10prom::gfp* | this work | 11 |
| OH15385 |  | *otIs684* | *srd-10prom::rab-3::gfp::unc-10_3'utr; unc-122prom::gfp* | this work | 2 |
| BC15959 | *dpy-5(e907)* | *sEx15959* | *srh-15prom::gfp; dpy-5(+)* | (McKay and Johnsen 2004) |  |
| CX3553 |  | *kyIs104* | *str-1prom::mcherry* | (Troemel *et al.* 1997) |  |
| CX3596 |  | *kyIs128* | *str-3prom::gfp* |  |  |
| OH14522 |  | *zfIs10* | *tdc-1prom::mcherry* | this work |  |
| OH13083 |  | *otIs576* | *unc-17fosmid::gfp; lin-44prom::yfp* | (Pereira *et al.* 2015) |  |
| OP173 |  | *wgIs173* | *unc-42* *^WRM0636dG10^fosmid::gfp* |  |  |
| OH14431 |  | *otIs638* | *unc-6fosmid::nls::yfp::h2b; pha-1(+); ttx-3prom::mcherry* | (Weinberg *et al.* 2018) |  |
| AQ3955 |  | *ljEx1067* | *WRM0616cB05 npr-11fosmid::sl2::gfp* |  |  |
| AX7054 |  | *dbEx1027* | *WRM0634bG04 frpr-8fosmid::SL2::GFP; unc-122prom::tagrfp* |  |  |
| OH15672 | *unc-42(e419)* | *otEx7280* | *WRM0636dG10 (unc-42 fosmid); unc-122prom::gfp* | this work | 3 |
| OH15764 | *unc-42(e419)* | *otEx7324* | *WRM0636dG10 (unc-42 fosmid); unc-122prom::gfp* | this work | 3 |
| OH15767 | *unc-42(e419)* | *otEx7325* | *WRM0636dG10 (unc-42 fosmid); unc-122prom::gfp* | this work | 3 |
| OH16894 | *hlh-34(syb2697); otIs696* | *otIs696* | *NeuroPAL* | This work | 12 |
| OH16894 | *hlh-34(syb2697);* lstEx6[pdf-2::GFP] | lstEx6 | *pdf-2prom::GFP* | This work | 12 |

Footnotes:

1: As per cell identification with NeuroPAL, this reporter is expressed in AVH and was previously misidentified to be expressed in AVJ (Cunningham et al., 2012).

2: To generate the *rab-3* transgenic strains *otIs684, otIs681, otIs680, otIs676, otIs677, otIs679,* and *otIs675*, the 1.2kb *srd-10^prom^* (*otIs684*) or the 1.7kb *nrp-9^prom^* (*otIs681, otIs680*) or the 234bp *flp-7^prom^* (*otIs676, otIs677, otIs679, otIs675*) was cloned to replace the *unc-30^prom^* in a plasmid kindly provided by John Kerk containing the *unc-30^prom^*, *gfp*, the *rab-3* cDNA, and the *unc-10 3’utr*. These plasmids were injected at 50ng/ul along with 50ng/ul of the *unc-122^prom^::gfp* injection marker. The extrachromosomal arrays derived from these injections were then gamma irradiated for integration.

3: To create these strains, the WRM0636dG10 fosmid was linearized with Not-I and injected into *unc-42(e419)* at a concentration of 15ng/ul, along with the *unc-122^prom^::gfp* injection marker at a concentration of 5ng/ul and 100ng/ul of PvuII-digested bacterial genomic DNA.

4: To create these strains, the 2.2kb *cfi-1^prom^* was cloned by deletion of the pSH405 plasmid (Shaham and Bargmann 2002) to keep only part of the 2^nd^ intron. For the 448bp *des-2^prom^*, the unc-86 binding site was removed and cloned into pPD95.75 containing *tagrfp*. These plasmids were injected into *pha-1(e2123)* along with a *pha-1(+)* rescuing array. The *cfi-1^prom^* plasmid was injected at 35ng/ul, the *des-2^prom^* plasmid was injected at 25ng/ul, and the *pha-1(+)* rescuing array was injected at 50ng/ul.

5: To create these strains, the 3.1kb *flp-18^prom^* was cloned from MVC12 (Park *et al.* 2011) into pPD95.75 containing *tagrfp*. This plasmid was injected at 50ng/ul. The extrachromosomal arrays derived from these injections were then gamma irradiated for integration.

6: To create this strain, the 2.7kb *sra-11^prom^* was cloned into pPD95.75 containing *wrmscarlet*. This plasmid was injected at 50ng/ul along with 4ng/ul of the inx-6^prom^::tagFRP coinjection marker (Bhattacharya and Hobert 2019). The extrachromosomal arrays derived from these injections were then gamma irradiated for integration.

7: To create these *unc-42* rescuing strains, pFG263 (a plasmid containing a 1.2kb *nmr-1^prom^*, *gfp*, and the *unc-42* cDNA in pPD49.26 (from the Fire vector kit)) was injected at 50ng/ul along with 50ng/ul of the *ttx-3^prom^::mcherry* injection marker.

8: To create these strains, the 1.2kb *nmr-1^prom^* was cloned to replace the *inx-18^prom^* in pPW3 (a plasmid kindly provided by Peter Weinberg containing an *inx-18^prom^*, the *unc-6* cDNA, and *nls::yfp::h2b* in pPD95.75 (from the Fire vector kit)). This plasmid was then injected at a concentration of 50ng/ul along with 50ng/ul of the *ttx-3^prom^::mcherry* injection marker.

9: *ot868 [unc-42::tagrfp::3Xflag]* was engineered using the procedure described in (Dickinson *et al.* 2015). The fluorescent tag was added to the C-terminus of UNC-42 separated by a short linker. The plasmid serving as a template for homologous recombination was cloned using Gibson and included 700 bp homology arms. Recombinants were verified by DNA sequencing.

10: *ot986 [unc-42::gfp]* was engineered using the procedure described in (Dokshin *et al.* 2018). The fluorescent tag was added to the C-terminus of UNC-42 separated by a short linker. The asymmetric dsDNA sequence serving as a template for homologous recombination was made by PCR and included 120bp homology arms. Recombinants were verified by DNA sequencing.

11: *otIs663 [srd-10^prom^::gfp]* was made by integration of *Ex [srd-10^prom^::GFP]* generated in the Ferkey lab, by gamma irradiation.

12: *hlh-34(syb2697)* is a 410bp deletion in the *hlh-34* locus created by CRISPR/Cas9 genome editing.

**Bibliography**

Aurelio, O., D. H. Hall and O. Hobert, 2002 Immunoglobulin-domain proteins required for maintenance of ventral nerve cord organization. Science 295**:** 686-690.

Barrios, A., R. Ghosh, C. Fang, S. W. Emmons and M. M. Barr, 2012 PDF-1 neuropeptide signaling modulates a neural circuit for mate-searching behavior in C. elegans. Nat Neurosci 15**:** 1675-1682.

Bhattacharya, A., U. Aghayeva, E. G. Berghoff and O. Hobert, 2019 Plasticity of the Electrical Connectome of C. elegans. Cell 176**:** 1174-1189 e1116.

Bhattacharya, A., and O. Hobert 2019 A new anterior pharyngeal region specific fluorescent co-transformation marker, pp. microPublication Biology.

Brenner, S., 1974 The genetics of Caenorhabditis elegans. Genetics 77**:** 71-94.

Chuang, C. F., M. K. Vanhoven, R. D. Fetter, V. K. Verselis and C. I. Bargmann, 2007 An innexin-dependent cell network establishes left-right neuronal asymmetry in C. elegans. Cell 129**:** 787-799.

Cohen, M., V. Reale, B. Olofsson, A. Knights, P. Evans *et al.*, 2009 Coordinated regulation of foraging and metabolism in C. elegans by RFamide neuropeptide signaling. Cell Metab 9**:** 375-385.

Consortium, C. e. D. M., 2012 large-scale screening for targeted knockouts in the Caenorhabditis elegans genome. G3 (Bethesda) 2**:** 1415-1425.

Dickinson, D. J., A. M. Pani, J. K. Heppert, C. D. Higgins and B. Goldstein, 2015 Streamlined Genome Engineering with a Self-Excising Drug Selection Cassette. Genetics 200**:** 1035-1049.

Dokshin, G. A., K. S. Ghanta, K. M. Piscopo and C. C. Mello, 2018 Robust Genome Editing with Short Single-Stranded and Long, Partially Single-Stranded DNA Donors in. Genetics 210**:** 781-787.

Hallam, S., E. Singer, D. Waring and Y. Jin, 2000 The C. elegans NeuroD homolog cnd-1 functions in multiple aspects of motor neuron fate specification. Development 127**:** 4239-4252.

Hart, A. C., J. Kass, J. E. Shapiro and J. M. Kaplan, 1999 Distinct signaling pathways mediate touch and osmosensory responses in a polymodal sensory neuron. J Neurosci 19**:** 1952-1958.

Howell, K., J. G. White and O. Hobert, 2015 Spatiotemporal control of a novel synaptic organizer molecule. Nature 523**:** 83-87.

Janssen, T., S. J. Husson, E. Meelkop, L. Temmerman, M. Lindemans *et al.*, 2009 Discovery and characterization of a conserved pigment dispersing factor-like neuropeptide pathway in Caenorhabditis elegans. J Neurochem 111**:** 228-241.

Jin, X., N. Pokala and C. I. Bargmann, 2016 Distinct Circuits for the Formation and Retrieval of an Imprinted Olfactory Memory. Cell 164**:** 632-643.

Kim, K., and C. Li, 2004 Expression and regulation of an FMRFamide-related neuropeptide gene family in Caenorhabditis elegans. J Comp Neurol 475**:** 540-550.

McKay, S. J., and R. K. Johnsen, J. Asano, J. Baillie, D.L. Chan, S. Dube, N. Fang, L. Goszczynski, B. Ha, E. Halfnight, E. Hollebakken, R. Huang, P. Hung, K. Jensen, V. Jones, S.J.M. Kai, H. Li, D. Mah, A. Marra, M. Mcghee, J. Newbury, R. Pouzyrev, A. Riddle, D.L. Sonnhammer, E. Tian, H. Tu, D. Tyson, J.R. Vatcher, G. Warner, A. Wong, K. Zhao, Z. Moerman, D.G., 2004 Gene Expression Profiling of Cells, Tissues, and Developmental

Stages of the Nematode C. elegans, pp. Cold Spring Harbor Symposia on Quantitative Biology.

Park, J., P. L. Knezevich, W. Wung, S. N. O'Hanlon, A. Goyal *et al.*, 2011 A conserved juxtacrine signal regulates synaptic partner recognition in Caenorhabditis elegans. Neural Dev 6**:** 28.

Pereira, L., P. Kratsios, E. Serrano-Saiz, H. Sheftel, A. E. Mayo *et al.*, 2015 A cellular and regulatory map of the cholinergic nervous system of C. elegans. Elife 4.

Pirri, J. K., A. D. McPherson, J. L. Donnelly, M. M. Francis and M. J. Alkema, 2009 A tyramine-gated chloride channel coordinates distinct motor programs of a Caenorhabditis elegans escape response. Neuron 62**:** 526-538.

Schroeder, N. E., R. J. Androwski, A. Rashid, H. Lee, J. Lee *et al.*, 2013 Dauer-specific dendrite arborization in C. elegans is regulated by KPC-1/Furin. Curr Biol 23**:** 1527-1535.

Schwarz, V., J. Pan, S. Voltmer-Irsch and H. Hutter, 2009 IgCAMs redundantly control axon navigation in Caenorhabditis elegans. Neural Dev 4**:** 13.

Sengupta, P., J. H. Chou and C. I. Bargmann, 1996 odr-10 encodes a seven transmembrane domain olfactory receptor required for responses to the odorant diacetyl. Cell 84**:** 899-909.

Serrano-Saiz, E., R. J. Poole, T. Felton, F. Zhang, E. D. de la Cruz *et al.*, 2013 Modular Control of Glutamatergic Neuronal Identity in C. elegans by Distinct Homeodomain Proteins. Cell 155**:** 659-673.

Shaham, S., and C. I. Bargmann, 2002 Control of neuronal subtype identity by the C. elegans ARID protein CFI-1. Genes Dev 16**:** 972-983.

Stefanakis, N., I. Carrera and O. Hobert, 2015 Regulatory Logic of Pan-Neuronal Gene Expression in C. elegans. Neuron 87**:** 733-750.

Troemel, E. R., B. E. Kimmel and C. I. Bargmann, 1997 Reprogramming chemotaxis responses: sensory neurons define olfactory preferences in C. elegans. Cell 91**:** 161-169.

Vidal, B., A. Santella, E. Serrano-Saiz, Z. Bao, C. F. Chuang *et al.*, 2015 C. elegans SoxB genes are dispensable for embryonic neurogenesis but required for terminal differentiation of specific neuron types. Development 142**:** 2464-2477.

Voelker, L., B. Upadhyaya, D. M. Ferkey, S. Woldemariam, N. D. L'Etoile *et al.*, 2019 INX-18 and INX-19 play distinct roles in electrical synapses that modulate aversive behavior in Caenorhabditis elegans. PLoS Genet 15**:** e1008341.

Wadsworth, W. G., H. Bhatt and E. M. Hedgecock, 1996 Neuroglia and pioneer neurons express UNC-6 to provide global and local netrin cues for guiding migrations in C. elegans. Neuron 16**:** 35-46.

Wang, X., W. Zhang, T. Cheever, V. Schwarz, K. Opperman *et al.*, 2008 The C. elegans L1CAM homologue LAD-2 functions as a coreceptor in MAB-20/Sema2 mediated axon guidance. J Cell Biol 180**:** 233-246.

Weinberg, P., M. Berkseth, D. Zarkower and O. Hobert, 2018 Sexually Dimorphic unc-6/Netrin Expression Controls Sex-Specific Maintenance of Synaptic Connectivity. Curr Biol 28**:** 623-629 e623.
