# Supplementary material for: The Prop1-like homeobox gene unc-42 specifies the identity of synaptically connected neurons
Source: eLife. 2021 Jun 24;10:e64903. doi: 10.7554/eLife.64903 (PMC8225392; doi:10.7554/eLife.64903)
Supplement: Supplementary file 6. [file elife-64903-supp6.docx]

**Supplementary File 6: Neuron identification in electron micrographs of *unc-42(e270)***

|  | **Axon Start Location** | **Axon End Location(s)** | **Nerve Ring Position** | **Additional Feature(s) Supporting Identification** |
| --- | --- | --- | --- | --- |
| AINL | left sublateral commisure | right ventral ganglion | outer and posterior | adjacent to AINR at the dorsal midline |
| AINR | right sublateral commisure | left ventral ganglion | outer and posterior | adjacent to AINL at the dorsal midline |
| ALA | cell body | right and left sublaterals | posterior |  |
| ALML | left sublateral commisure | left ventral ganglion | inner and posterior | microtubule-filled neurite on the left, located in close proximity to the hypodermis and cuticle |
| ALMR | right sublateral commisure | right ventral ganglion | inner and posterior | microtubule-filled neurite on the right, located in close proximity to the hypodermis and cuticle |
| AVAL | cell body | right ventral ganglion | anterior | adjacent to AVAR at the dorsal midline |
| AVAR | cell body | left ventral ganglion | anterior | adjacent to AVAL at the dorsal midline |
| AVBL | left ventral ganglion | right ventral ganglion | posterior | close association with AVJL and AVJR |
| AVER | cell body | left ventral ganglion | anterior |  |
| AVHL | left sublateral commisure | right ventral ganglion | middle and posterior | adjacent to AVHR at the dorsal midline, close and posterior association with AVJL |
| AVHR | right sublateral commisure | left ventral ganglion | middle and posterior | adjacent to AVHL at the dorsal midline, close and posterior association with AVJR |
| AVJL | left sublateral commisure | right ventral ganglion | middle and posterior | adjacent to AVJR at the dorsal midline, close and anterior association with AVHL |
| AVJR | right sublateral commisure | left ventral ganglion | middle and posterior | adjacent to AVJL at the dorsal midline, close and anterior association with AVHR |
| BAGL | cell body | right ventral ganglion | outer | adjacent to BAGR at the dorsal midline |
| BAGR | cell body | left ventral ganglion | outer | adjacent to BAGL at the dorsal midline |
| OLLR | right labial commissure | right ventral ganglion and dorsal midline | inner |  |
| RMER | cell body | right nerve ring (premature) and left ventral ganglion | inner | inner ventral placement of axon at the base of the nerve ring |
| SAAVL | left sublateral commisure | right ventral ganglion | middle | axon projects into the sublateral cord in the head |
| SMDVL | cell body | right ventral ganglion | middle |  |
